# The promoting effect of modified Dioscorea pills on vascular remodeling in chronic cerebral hypoperfusion via the Ang/Tie signaling pathway

**DOI:** 10.1515/tnsci-2022-0302

**Published:** 2023-08-23

**Authors:** Guiying Kuang, Zhigang Shu, Chunli Zhu, Hongbing Li, Cheng Zhang

**Affiliations:** Neurological Department, Wuhan Red Cross Hospital, Wuhan, Hubei Province, 436015, China; Neurological Department, Ezhou Central Hospital, Ezhou, Hubei Province, 436000, China; Emergency Department, The First People’s Hospital of Guiyang, Guiyang, Guizhou Province, 550002, China

**Keywords:** modified Dioscorea pills, chronic cerebral hypoperfusion, Ang/Tie signaling pathway, microvessel density

## Abstract

**Objective:**

The objective of this study was to investigate the effect of modified Dioscorea pills (MDP) on microcirculatory remodeling in the hippocampus of rats with chronic cerebral hypoperfusion (CCH) through the angiopoietin (Ang)/tyrosine kinase receptor tyrosine kinase with immunoglobulin-like and EGF-like domains (Ang receptor) 2 (Tie-2) signaling pathways, which may underlie the cognitive improvement observed in CCH rats.

**Methods:**

Forty male Sprague–Dawley rats raised under specific pathogen-free conditions were randomly divided into three groups: control group (10 rats), model group (15 rats), and MDP group (15 rats). The rats in the model group and MDP group underwent bilateral common carotid artery occlusion using the 2-vessel occlusion (2-VO) method to induce CCH. Rats in the control group underwent the same surgical procedures as those in the model group, except for ligation and occlusion of the carotid arteries. After 1 week of 2-VO, rats in the MDP group were administered MDP condensed decoction intragastrically at a dose of 1 ml/100 g body weight (prepared by the Preparation Room of Hubei Provincial Hospital of Traditional Chinese Medicine) for 45 days, while rats in the other two groups received normal saline intragastrically with the same dose and duration as the MDP group. After the intervention, all rats were euthanized, and brain perfusion was performed to obtain the hippocampal tissue for analysis. Immunohistochemical staining for CD43 was performed to assess microvessel density (MVD); western blot and the reverse transcription-polymerase chain reaction (RT-PCR) were used to analyze the expression of proteins and genes in angiopoietin-1 (Ang-1), angiopoietin-2 (Ang-2), Tie-2, and vascular endothelial growth factor (VEGF) proteins and genes in the hippocampal tissue and compute the Ang-1/Ang-2 ratio.

**Results:**

MDP treatment reduced neuronal loss and promoted restoration of the damaged hippocampal structure in CCH rats. The model group showed significantly higher MVD (14.93 ± 1.92) compared to the control group (5.78 ± 1.65) (*P* < 0.01), whereas MDP treatment further increased MVD (21.19 ± 2.62). Western blot and RT-PCR analysis revealed that CCH significantly increased the expression of Ang-1, Ang-2, Tie-2, and VEGF proteins and genes, while MDP treatment further significantly upregulated the expression of these proteins and genes. In addition, MDP significantly elevated the gene and protein expression of the Ang-1/Ang-2 ratio compared to the control group (*P* = 0.041, *P* = 0.029).

**Conclusion:**

CCH induces microvascular neogenesis in the hippocampus, and MDP promotes angiogenesis and microcirculation remodeling in CCH rats via the Ang/Tie signaling pathway, which may be an important mechanism for its restorative effects on hippocampal perfusion and improvement of cognitive function in CCH rats.

## 1 Introduction

Chronic cerebral hypoperfusion (CCH) is a condition that is becoming more prevalent and severe in the elderly population, likely due to aging and the progression of atherosclerosis. CCH has been strongly associated with cognitive dysfunction and other neuropsychiatric diseases. One of the underlying mechanisms of CCH is the increased levels of reactive oxygen species, which can lead to neuronal damage and neuroinflammation [1]. This neuroinflammation has been implicated in various neuropsychiatric diseases such as vascular dementia [2], chronic epilepsy [3], and bipolar disorder [4]. Currently, the treatment for CCH primarily focuses on protecting neurons and improving cerebral perfusion, as these are important factors in mitigating the detrimental effects of CCH on brain health.

The rat model of CCH can be induced using the 2-vessel occlusion (2-VO) method, which has been shown to cause neuronal loss in the cerebral cortex and hippocampus [5]. The 2-VO model is commonly used to study and intervene in CCH. Previous research has demonstrated that modified Dioscorea pills (MDP), a well-known prescription for treating vascular dementia, can improve episodic memory and cognitive impairment in rats with CCH induced by 2-VO. The beneficial effects of MDP are attributed to its ability to inhibit inflammation, reduce neuronal damage, promote glial proliferation, enhance synaptic protein expression and synaptic plasticity, and upregulate the expression of key genes such as GAP-43 and vascular endothelial growth factor (VEGF) in the hippocampus [6]. In recent years, there has been a shift in focus from solely studying neurons to considering the comprehensive role of neurovascular units in neurological diseases [7,8]. Cerebral microvessels play a critical role in maintaining normal brain function, and further investigation is required to determine whether MDP can improve CCH while protecting and restoring the neural structure of the hippocampus.

Studies have shown that hypoxia or inflammation can lead to increased expression of angiogenic factors such as VEGF, bFGF, and PDGF, which bind to their corresponding receptors on the cell membrane of vascular endothelial cells, triggering downstream signaling cascades and promoting angiogenesis. This process involves the dissolution of the vascular basement membrane by extracellular matrix metalloproteinases, leading to the dissociation of vascular endothelial cells and the formation of new blood vessels [9]. Over time, these newly formed microvessels integrate into the existing vascular system and mature into stable blood vessels. The angiopoietin (Ang)/tyrosine kinase receptor tyrosine kinase with immunoglobulin-like and EGF-like domains (Ang receptor) 2 (Tie-2) and VEGF/vascular endothelial growth factor receptor 2 signaling pathways are known to be involved in angiogenesis and maintenance of vascular stability [10]. Generally, angiopoietin-1 (Ang-1) ligands act via the Tie-2 receptor, playing a stabilizing role in vascular endothelium. On the other hand, angiopoietin-2 (Ang-2) blocks Ang1 activation of Tie-2, leading to endothelial cell instability and promoting vascular remodeling [11,12]. Further investigation is required to explore how MDP may interfere with hippocampal vascular remodeling in the context of CCH and improve cognitive function via the Ang/Tie signaling pathway. This could shed light on the mechanism by which MDP exerts its beneficial effects on CCH and cognitive function, and potentially provide insights for developing novel therapeutic strategies for CCH and related neurovascular disorders.

## 2 Materials and methods

### 2.1 Animals

Male Sprague–Dawley rats (40 in total) with specific-pathogen-free status were purchased from the Hubei Provincial Academy of Preventive Medicine (License number: SCXK(Hubei)2015-0018). The rats had an average body weight of approximately 280 ± 20 g and were housed in the Experimental Animal House of Guizhou Medical University under standard laboratory conditions, including an ambient temperature of 23 ± 1°C, relative humidity of 50 ± 10%, and a 12 h light/dark cycle. They were provided with free access to food and water. The rats were acclimated to the housing conditions for 1 week prior to the experimental procedures.

### 2.2 Preparation of the CCH model and intervention measures

A total of 40 rats were randomly assigned to three groups: control group (*n* = 10), model group (*n* = 15), and MDP group (*n* = 15), using a random number table. The CCH models, consisting of the model group and MDP group, were induced by bilateral common carotid artery occlusion (2-VO) wherein the rats’ left and right common carotid arteries were ligated separately, with a 1 week interval. Rats in the control group underwent the same surgical procedures as the model groups, except for the ligation and occlusion of the bilateral common carotid arteries. During surgery, the rectal temperature of the rats was maintained at 37.0–37.9°C using a heating pad to prevent brain injury due to low temperature. After approximately 2–4 min, all animals regained consciousness and were provided with free access to food and water. After 1 week of 2-VO surgery, rats in the MDP group were administered MDP condensed decoction intragastrically at a dose of 1 ml/100 g body weight (prepared by the Preparation Room of Hubei Provincial Hospital of Traditional Chinese Medicine) for 45 days, while rats in the other two groups were treated with normal saline intragastrically at the same dose and duration as the MDP group.

### 2.3 Sampling

After the gavage intervention, rats were euthanized, and brain tissues were obtained by perfusion sampling and fresh sampling. Perfusion sampling was conducted as follows: five rats from each group were deeply anesthetized with isoflurane inhalation, as previously described. Their hearts were exposed, and perfusion needles were inserted through the apex into the left ventricle and then into the aorta. The right atrial appendage was quickly cut, and 250 ml of normal saline was perfused through the perfusion needles. As clear perfusate flowed out, the rats’ tails straightened, trembling limbs ceased, and the livers gradually turned white. Subsequently, 250 ml of 4% paraformaldehyde was perfused successively. After approximately 40 min, the rats were decapitated, and the hippocampus was isolated and harvested. The harvested tissues were then fixed in 4% paraformaldehyde buffer solution for 12–24 h, followed by conventional dehydration, paraffin embedding, and staining with hematoxylin and eosin (HE) and immunohistochemical staining. Fresh sampling was performed for reverse transcription-polymerase chain reaction (RT-PCR) and western blot detection, involving five rats from each group. The steps for fresh sampling were as follows: rats were deeply anesthetized with isoflurane inhalation and then decapitated, and brain tissues were quickly stripped for RT-PCR detection.

### 2.4 Histopathological observation

Paraffin sections of the hippocampus were obtained for HE staining in order to observe the pathological characteristics of the CA1, CA3, and DG regions. The hippocampal tissue was dehydrated, rendered transparent, and embedded in paraffin. Subsequently, the tissue was cut into 4 µm slices, which were then baked, dewaxed, and stained with HE for subsequent microscopic observation using an Olympus biological microscope (Type BX53) at magnifications of 100× and 400×.

### 2.5 Immunohistochemistry and microvessel density (MVD) analysis

Immunohistochemistry and MVD detection were performed using the hippocampus of three rats from each group. Paraffin sections were prepared following the same procedure as for histopathological analysis. After antigen retrieval and blocking of endogenous peroxidase, the first antibodies (mouse anti-CD43) were added to the hippocampus paraffin sections. Subsequently, IgG polymers of horseradish peroxidase-labeled secondary antibody and freshly prepared DAB color solution were successively added to these sections. Finally, the sections were counterstained with Harris hematoxylin.

For the analysis of MVD, CD43-labeled images were collected. According to the corrected Weidner method, any single endothelial cell or cell clusters stained by anti-CD43, as long as they have a clear boundary with the surrounding microvessels or other connecting tissues, regardless of whether a lumen has formed, were counted as a microvessel, excluding vessels with smooth muscle walls or lumen diameters greater than eight times the diameter of erythrocytes. Each sample was first observed at 100× magnification to select three spots with the highest number of microvessels as “hot spots.” Then, the microvessels were counted at high magnification (400×) from the hot spots, and the average number of microvessels was calculated as MVD.

### 2.6 Western blot

To the hippocampus tissue was added RIPA lysate (Meilunbio, product no.: MA0151) to extract the total proteins of the hippocampus. By diluting and boiling, the extracted proteins were denatured prior to electrophoresis on the gel, and these proteins were transferred into the PVDF membrane (Genscript, product no. L00791C). They were sealed and reacted with primary antibodies (rabbit anti-GAPDH, 37 kD, Hangzhou Xianzhi Biological Co., Ltd., product no. AB-P-R 001, 1:10,000 dilution; rabbit anti-Ang-1 [70 kD], Wuhan Sanying, product no. 23302-1-AP, 1:10,000 dilution; rabbit anti-Ang-2, 75 kD, product number: ABclonal A0698, diluted 1:10,000; rabbit anti-tie2126 kD, ABclonal, product number A7222, diluted at 1:10,000; rabbit anti-VEGF, 45 kD, Wuhan Sanying, product number: 19003-1-AP, 1:10,000 dilution). Afterward, HRP-labeled secondary antibodies (HRP-labeled sheep antimouse secondary antibody, Wuhan Doctor Bioengineering Co., Ltd., product number: BA1051, 1:10,000 dilution) were added and the films were developed, fixed, and processed. Finally, the films were analyzed with ipp6.0 software to determine the film grayscale value using GAPDH as an inner reference.

### 2.7 RT-PCR

Hippocampal tissues weighing 100 mg were extracted to obtain RNA using the Trizol method. The extracted RNA was then reverse-transcribed into cDNA, followed by the addition of designed primers for real-time fluorescence quantitative reaction using the SYBR Green dye method. Amplification and melting curves were drawn, and the relative expression of target genes such as Ang-1, Ang-2, Tie-2, and VEGF was calculated using the QPCR algorithm (relative quantification, 2⁻^ΔΔ*C*t^), with GAPDH as an internal reference (Table 1).

**Table 1:** Primer sequence table

Gene	Primer	Sequence (5′–3′)	PCR products (bp)
Rat GAPDH	Forward	ACAGCAACAGGGTGGTGGAC	253
	Reverse	TTTGAGGGTGCAGCGAACTT	
Rat Ang-1	Forward	AATGTGCCTACACTTTCA	345
	Reverse	GATTTAGTACCTGGGTCTC	
Rat Ang-2	Forward	GTGGCAGATTGTTTTCCT	231
	Reverse	TGCACTGAGTCGTCGTAG	
Rat Tie-2	Forward	GCTCTGGGAGATCGTTAGCT	151
	Reverse	CTCCCTCCAGCATTGTCTCA	
Rat VEGF	Forward	CCCACGACAGAAGGGGAGCA	161
	Reverse	CGCATTAGGGGCACACAGGAC	

### 2.8 Statistics

All data obtained in this experiment, except for HE staining, are presented as quantitative values expressed as mean ± standard deviation (mean ± SD). Statistical analysis was performed using SPSS 19.0 software. *T*-test was used for intergroup comparisons, and one-way ANOVA was used for comparisons among multiple groups. A significance level of *P* < 0.05 was considered statistically significant, while *P* < 0.01 was considered extremely statistically significant.


**Ethical approval:** The research related to animals’ use complied with all the relevant national regulations and institutional policies for the care and use of animals. All animal handling and experimental protocols were conducted in accordance with the Guidance Suggestions for the Care and Use of Laboratory Animals, formulated by the Ministry of Science and Technology of the People’s Republic of China. The study was approved by the Committee on Ethics of the First People’s Hospital of Guiyang (Guizhou, China), with ethical approval number 2022007.

## 3 Results

### 3.1 Pathology observation

**Figure 1:**
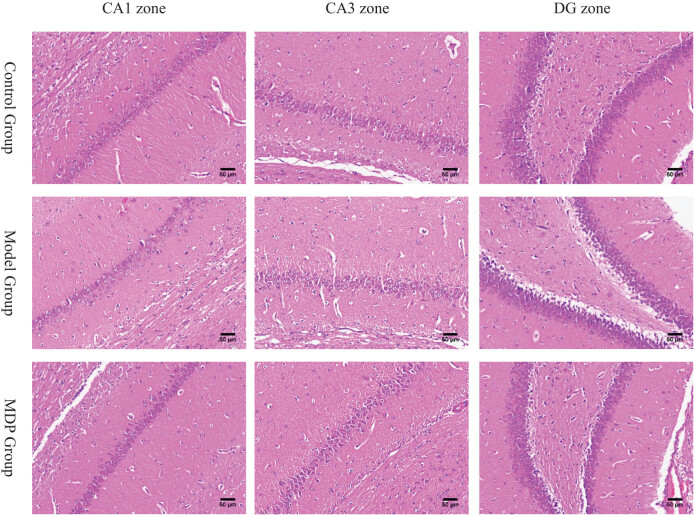
The pathology of CA1, CA3, and DG zones in the hippocampus among the three groups. CCH induced neurons loss, white matters loosen and disarrange in CA1, CA3, and DG zones in the hippocampus. MDP significantly restored them. Using Image J software, we calculated that the total neurons in the CA1 zone are, respectively, 155 ± 21, 110 ± 18, 182 ± 36 per slice (400 times magnetite). There are significant differences between the two groups (*P* < 0.05).

HE staining revealed that compared to the control group, the model group showed a significant loss of neurons with disordered arrangement in the CA1, CA3, and DG regions of the hippocampus. The radiation layer appeared loose with pale staining and showed multiple cracks in the CA1 region. Many disarranged cells infiltrated the white matter of the CA3 region, and a large blank gap was observed between the cortex and subcortical in the DG region. However, in the MDP group, these abnormal pathological findings were significantly restored, with evident regeneration of neurons in the CA1, CA3, and DG regions (Figure 1).

### 3.2 Immunohistochemistry

**Figure 2:**
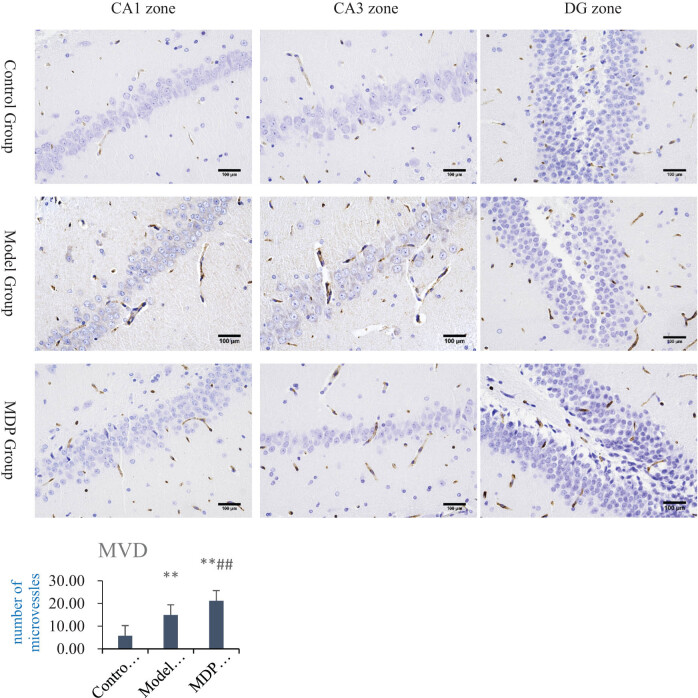
Immunohistochemistry CD43 staining in the hippocampus and MVD analysis. MVD in the model group (14.93 ± 1.92), control group (5.78 ± 1.65), and the MDP group (21.19 ± 2.62). **P < 0.01 vs control group; ^##^ *P* < 0.01 vs the model group.

CCH induced by 2-VO resulted in evident neovascularization in the hippocampus. Compared with the control group, the MVD in the model group (14.93 ± 1.92) was significantly higher (*P* < 0.01) than that in the control group (5.78 ± 1.65). Furthermore, the MVD in the MDP group (21.19 ± 2.62) was significantly higher (*P* < 0.01, *P* < 0.01) than that of both the model group and control group, indicating that MDP further promotes microvascular regeneration (Figure 2).

### 3.3 Western blot

**Figure 3:**
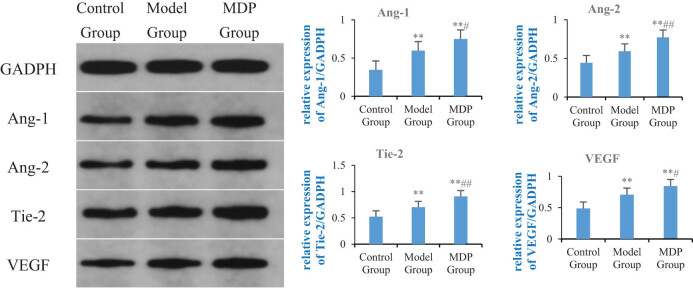
Expressions of Ang-1, Ang-2, Tie-2, and VEGF in the hippocampus among the three groups. Using GAPDH as the inner reference, the relative expression levels of Ang-1, Ang-2, Tie-2, and VEGF in the hippocampus form are calculated. The left image shows the immunoblotting of GAPDH, Ang-1, Ang-2, Tie-2, and VEGF, while the right bar graph, respectively, shows the relative expression levels of Ang-1, Ang-2, Tie-2, and VEGF in each group of rats. **P < 0.01 vs control group; ^#^ *P* < 0.05 vs model group; ^##^ *P* < 0.01 vs model group.

The expression of Ang-1 in the model group was significantly increased compared to the control group (0.597 ± 0.090 vs 0.345 ± 0.058, *P* = 0.005), and MDP further upregulated the expression of Ang-1 (0.750 ± 0.060), with significant differences observed when compared to both the control group and the model group (*P* < 0.001, *P* = 0.039). Similarly, the expression of Ang-2 in the model group was significantly increased compared to the control group (0.592 ± 0.035 vs 0.442 ± 0.068, *P* = 0.007), and MDP further upregulated the expression of Ang-2 (0.771 ± 0.017), with significant differences observed when compared to both the control group and the model group (*P* < 0.001, *P* = 0.003). Furthermore, the expression of Tie-2 in the model group was significantly increased compared to the control group (0.703 ± 0.025 vs 0.524 ± 0.044, *P* < 0.001), and MDP further upregulated the expression of Tie-2 (0.909 ± 0.019), with significant differences observed when compared to both the control group and the model group (*P* < 0.001, *P* < 0.001). Finally, the expression of VEGF in the model group was significantly increased compared to the control group (0.706 ± 0.075 vs 0.486 ± 0.030, *P* = 0.002), and MDP further upregulated the expression of VEGF (0.844 ± 0.034), with significant differences observed when compared to both the control group and the model group (*P* < 0.001, *P* = 0.016) (Figure 3).

### 3.4 RT-PCR

**Figure 4:**
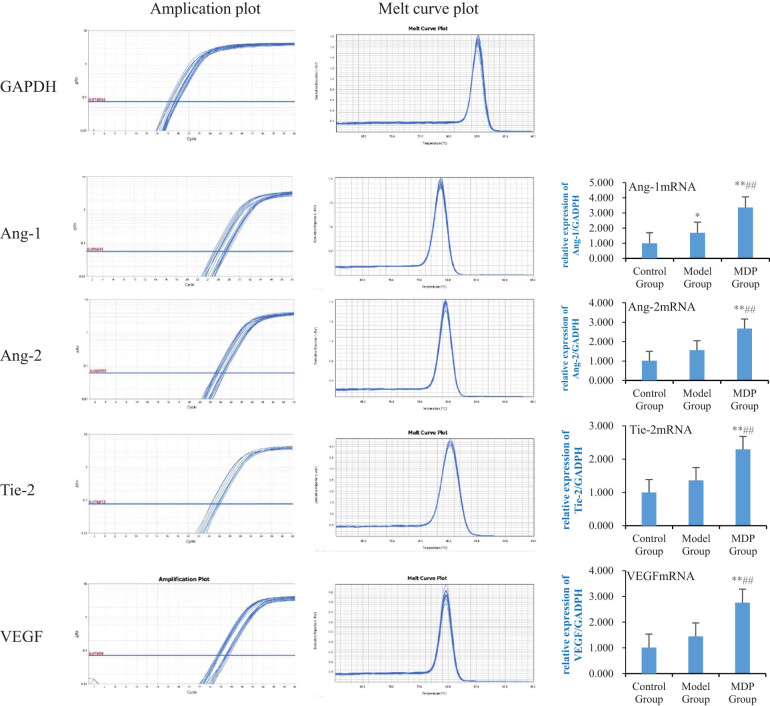
Expressions of Ang-1 mRNA, Ang-2 mRNA, Tie-2 mRNA, and VEGF mRNA in the hippocampus among the three groups. Using GAPDH as the inner reference, the relative expression levels of Ang-1 mRNA, Ang-2 mRNA, Tie-2 mRNA, and VEGF mRNA in the hippocampus form are calculated. The left image shows the amplification and fusion curves of GAPDH, Ang-1, Ang-2, Tie-2, and VEGF genes. The right bar graph shows the relative expression levels of Ang-1 mRNA, Ang-2 mRNA, Tie-2 mRNA, and VEGF mRNA in each group of rats. **P < 0.01 vs control group; *P < 0.05 vs control group; ^##^ *P* < 0.01 vs model group.

The expression of Ang-1 mRNA in the model group was significantly increased compared to the control group (1.691 ± 0.488 vs 0.992 ± 0.034, *P* = 0.032), and MDP further upregulated it (3.358 ± 0.596), with significant differences observed compared to both the control group and the model group (*P* <0.001, *P* <0.001). The expression of Ang-2 mRNA in the model group showed a tendency to increase compared to the control group but did not reach statistical significance (1.560 ± 0.491 vs 1.011 ± 0.016, *P* = 0.203). However, MDP further upregulated the expression of Ang-2 mRNA (2.673 ± 0.804), with significant differences observed compared to both the control group and the model group (*P* < 0.001, *P* = 0.002). Similarly, the expression of Tie-2 mRNA in the model group showed an increasing trend compared to the control group but did not reach statistical significance (1.362 ± 0.297 vs 1.001 ± 0.002, *P* = 0.095). Nevertheless, MDP further upregulated the expression of Tie-2 mRNA (2.294 ± 0.443), with significant differences observed compared to both the control group and the model group (*P* < 0.001, *P* < 0.001). The expression of VEGF mRNA in the model group also showed a tendency to increase compared to the control group but did not reach statistical significance (1.447 ± 0.264 vs 1.012 ± 0.036, *P* = 0.219). However, MDP further upregulated the expression of VEGF mRNA (2.754 ± 0.797), with significant differences observed compared to both the control group and the model group (*P* < 0.001, *P* < 0.001) (Figure 4).

### 3.5 Ang-1/Ang-2 ratio

The Ang-1/Ang-2 ratio suggests that there are no significant differences in the gene expression ratio among the three groups (*F* = 2.576, *P* = 0.097) but MDP still significantly elevated the gene expression ratio of Ang-1/Ang-2 compared to the control group (*P* = 0.041). In terms of protein expression, in the physiological state (control group), the expression of Ang-1 is less than Ang-2, the ratio of Ang-1/Ang-2 is 0.779 ± 0.046, the interventions of CCH or MDP obviously increase the expression of Ang-1 much more than that of Ang-2, thus the ratios of Ang-1/Ang-2, respectively, reach 1.007 ± 0.113 and 0.972 ± 0.077. All reach significant differences when compared to that of the control group (*P* = 0.015, *P* = 0.029) but there is no significant difference between the model group and the MDP group (*P* = 0.627) (Table 2).

**Table 2:** Ratio of Ang-1/Ang-2 in gene or protein expressions among the three groups

	Control group	Model group	MDP group
Gene ratio	1.015 ± 0.296	1.092 ± 0.196	1.356 ± 0.458*
Protein ratio	0.779 ± 0.046	1.007 ± 0.113*	0.972 ± 0.077*

* *P* < 0.05 vs control group.

## 4 Discussion

In this study, the results of HE staining confirmed that MDP exerted neuroprotective and neural repair effects in the hippocampus, indicating its potential to improve blood supply in the hippocampus of CCH rats. This was further supported by CD43 immunohistochemistry staining and MVD analysis, which demonstrated that CCH induced spontaneous angiogenesis in the CA1, CA3, and DG regions of the hippocampus, and that MDP further enhanced the formation of microvessels. This suggests that the promotion of angiogenesis may be an important mechanism through which MDP improves blood supply in the hippocampus of CCH.

Angiogenesis and vascular remodeling are constant processes in the adult vascular system, adapting to the needs of blood supply and organ function by the communication between blood vessels and parenchymal cells [13]. Vascular integrity and remodeling are co-regulated by endothelial growth factors and inflammatory cytokines. The angiopoietin/tie (Ang/Tie) pathway is known to play a crucial role in regulating vascular stability and angiogenesis in physiological and pathological conditions, including improving blood supply to specific organs during inflammation [14]. Ang-1 is primarily produced by pericytes and platelets. Under physiological conditions, Ang-1 activates downstream signals by binding and phosphorylating Tie-2 receptors, promoting intercellular junctions and enhancing endothelial cell survival, thereby maintaining vascular stability and normal vascular function [15,16,17]. Ang-2, on the other hand, is mainly produced by endothelial cells and is physiologically expressed in vascular remodeling sites such as the ovaries, placenta, and uterus [18]. However, Ang-2 expression can be triggered by inflammatory mediators such as thrombin [19], hypoxia [20], and cancer [21] and is upregulated under pathological conditions such as macular edema, neuroinflammation, and sepsis [18,22]. Ang-2 acts as an antagonist of the Ang-1/Tie-2 axis, causing instability of blood vessels and rendering them susceptible to vascular endothelial growth factor A (VEGF). Ang-2 and VEGF synergistically drive vascular leakage, neovascularization, and inflammation [23,24]. Previous studies have shown that decreased Ang-2 expression and increased Ang-1 expression contribute to vascular remodeling in the ischemic brain [25]. In this study, regardless of gene or protein expression, Ang-1, Ang-2, Tie-2, and VEGF were upregulated in the model group, indicating that under CCH conditions, Ang-1, Tie-2, and VEGF were upregulated to meet the needs of the ischemic and inflammatory environments, promoting microvascular stability in the hippocampus. The upregulation of Ang-2 may be related to the remodeling of hippocampal blood vessels caused by hypoxia and inflammation. MDP further promotes significant upregulation of these modulating cytokines, especially Ang-1, to promote and maintain the stability of the existing blood vessels and the modification of removal of the defective blood vessels, thus improving blood supply in the hippocampus. In summary, Ang-1 plays a crucial role in maintaining the stability of the existing blood vessels and ensuring blood supply, while Ang-2 promotes the degradation of damaged blood vessels, creating conditions for the formation of new blood vessels. Together, they jointly contribute to improving blood supply under pathological conditions.

In terms of angiogenesis, Ang-1 activates Tie-2 to promote the migration of endothelial cells and facilitate the development of blood vessels in areas lacking blood vessels [26], thereby restoring cerebral perfusion in the ischemic area and preserving neurons in the ischemic penumbra from dying. Tie-2 also induces angiogenesis through independent mechanisms [27]. Additionally, in the presence of injury and hypoxia, upregulated VEGF enhances PI3K/AKT and MEK1/2/ERK1/2 signal transduction, leading to increased proliferation and migration of residual capillary endothelial cells in the ischemic penumbra [28]. VEGF is involved in almost all processes of angiogenesis, directly influencing the occurrence and development of angiogenesis and ultimately determining the outcomes of angiogenesis to some extent. In this study, Ang-1, Tie-2, and VEGF were found to be upregulated in the model group, indicating their important roles as regulatory factors in promoting angiogenesis. Consequently, the mean vessel density (MVD) in the model group was significantly higher than that in the control group. MDP treatment significantly increased the expression of these cytokines, suggesting that it is an important mechanism for promoting angiogenesis, improving hippocampal perfusion, and restoring cognitive function in CCH.

Tie-2 receptor is a tyrosine kinase receptor that is expressed in endothelial cells [29] and hematopoietic stem cells [30] and it binds to all four angiopoietins [31]. Oligomeric Ang-1 promotes the binding between Tie-2 and cells. Moreover, Tie-2 can also bind fibronectin, collagen, and vitronectin with high affinity, thereby anchoring Tie-2 to the extracellular matrix [32], which are important mechanisms through which Tie-2 promotes vascular stability. A high concentration (800 ng/mL) of Ang-2 can induce the phosphorylation of the Tie-2 receptor, leading to increased cell survival, proliferation, chemotaxis of brain capillary endothelial cells, and angiogenesis [33,34]. It can be observed that MDP promotes the upregulation of Ang-1, Ang-2, and Tie-2, which collectively enhance the stability of the existing blood vessels and the formation of new blood vessels. These mechanisms may explain why MDP significantly enhances MVD in CCH.

The increased levels of Ang-2 induced by hypoxia activate Tie-2, which leads to the detachment and migration of pericytes from the basement membrane [35], resulting in increased vascular permeability and leakage. Additionally, Ang-2 can interact with integrin β1 [36] or αvβ [37], disrupting endothelial stability and promoting vascular leakage independently of Tie-2. In the first 3 days after stroke, elevated Ang-2 levels were associated with detrimental vascular permeability, while high Ang-2 levels after 7 days were associated with microvascular stability and maturation [38]. Although long-term overexpression of Ang-2 alone can promote vascular degeneration [39], in this study, when Ang-2 levels were upregulated along with VEGF in CCH, Ang-2 actually promoted angiogenesis [40]. Moreover, Ang-2 promotes the differentiation of neural progenitor cells and independently mediates their migration through Tie-2 [41], indicating that Ang-2 not only promotes vascular remodeling but also facilitates neural remodeling in CCH. Therefore, Ang-2 is an important target for MDP to improve hippocampal blood supply and promote neural recovery in CCH.

From the analysis of the Ang-1/Ang-2 ratio, we can see that MDP not only greatly increases the expressions of Ang1/2, VEGF, and Tie-2 compared to the control group and the model group, it also increased the ratio of Ang-1/Ang-2 compared to the control group, with the increase in ratio of Ang-1.

Ang-2 can alleviate cerebral ischemia/reperfusion injury by maintaining the integrity of blood vessels and promoting perfusion [42]. Therefore, the high-level expressions of Ang1/2, VEGF, and Tie-2 may all contribute to the high MVD and hippocampal perfusion in CCH.

## 5 Conclusion

The Ang-Tie-2 signaling pathway is a crucial pathway that regulates vascular stability and angiogenesis, with its regulatory effects varying depending on its concentration and interactions with other cytokines. In CCH, MDP induces significant upregulation of Ang-1, Ang-2, Tie-2, and VEGF, promoting both the stability of the original hippocampal blood vessels and the regeneration of microvessels. This leads to the reconstruction of the hippocampal microcirculation network, increased hippocampal perfusion, and improved cognitive function in rats with CCH.
